# Lignin Precipitation and Fractionation from OrganoCat Pulping to Obtain Lignin with Different Sizes and Chemical Composition

**DOI:** 10.3390/molecules25153330

**Published:** 2020-07-22

**Authors:** Dennis Weidener, Arne Holtz, Holger Klose, Andreas Jupke, Walter Leitner, Philipp M. Grande

**Affiliations:** 1 Institute of Bio- and Geosciences, Plant Sciences, Forschungszentrum Jülich GmbH, 52425 Jülich, Germany; d.weidener@fz-juelich.de (D.W.); h.klose@fz-juelich.de (H.K.); 2 Institute of Technical and Macromolecular Chemistry (ITMC), RWTH Aachen University, Worringer Weg 1, 52074 Aachen, Germany; walter.leitner@cec.mpg.de; 3 Bioeconomy Science Center (BioSC), c/o Forschungszentrum Jülich, 52425 Jülich, Germany; Arne.Holtz@avt.rwth-aachen.de; 4 Fluid Process Engineering (AVT.FVT), RWTH Aachen University, Forckenbeckstraße 51, 52074 Aachen, Germany; Andreas.Jupke@avt.rwth-aachen.de; 5 Institute of Biology I, RWTH Aachen University, Worringer Weg 3, 52074 Aachen, Germany; 6 Max-Planck-Institute of Chemical Energy Conversion, Stiftstraße 34-36, 45470 Mülheim an der Ruhr, Germany

**Keywords:** lignin fractionation, OrganoCat pulping, lignin characterization, lignocellulose, biorefinery, β-*O*-4-linkage, antisolvent precipitation

## Abstract

Fractionation of lignocellulose into its three main components, lignin, hemicelluloses, and cellulose, is a common approach in modern biorefinery concepts. Whereas the valorization of hemicelluloses and cellulose sugars has been widely discussed in literature, lignin utilization is still challenging. Due to its high heterogeneity and complexity, as well as impurities from pulping, it is a challenging feedstock. However, being the most abundant source of renewable aromatics, it remains a promising resource. This work describes a fractionation procedure that aims at stepwise precipitating beech wood (*Fagus sp.*) lignin obtained with OrganoCat technology from a 2-methyltetrahydrofuran solution, using *n*-hexane and *n*-pentane as antisolvents. By consecutive antisolvent precipitation and filtration, lignin is fractionated and then characterized to elucidate the structure of the different fractions. This way, more defined and purified lignin fractions can be obtained. Narrowing down the complexity of lignin and separately valorizing the fractions might further increase the economic viability of biorefineries.

## 1. Introduction

Due to depleting fossil resources and reorientation towards renewable energy and resources, biorefining will become a key technology in the near future. Various concepts exist that aim at using biomass by converting it into usable fine chemicals, fuels, or food additives [1]. The most abundant biomass on the planet is lignocellulose, which is a prominent example in modern biorefinery concepts [2,3,4]. It mainly consists of three different polymers: cellulose, hemicelluloses, and lignin.

**Scheme 1:**
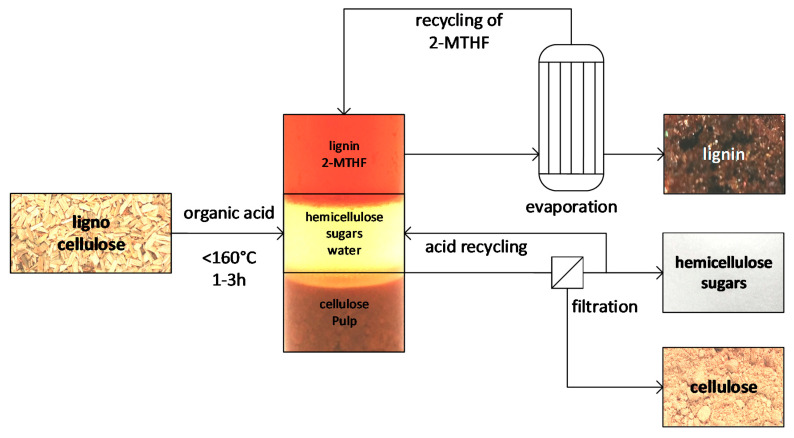
OrganoCat process for the fractionation of lignocellulose into its three main components: cellulose, hemicelluloses, and lignin [4,10,11].

Cellulose and hemicelluloses are homogeneous polysaccharides and their use for applications has already been broadly discussed [5]. Lignin, on the other hand, is a complex heterogeneous macromolecule, consisting mainly of the three aromatic subunits *p*-hydroxyphenyl (**H**), guaiacyl (**G**), and syringyl (**S**). These subunits are mainly linked via linkages such as β-*O*-4, β-β, and β-5 [6]. Due to the high heterogeneity of lignin, which is even enhanced by harsh process conditions in biomass pulping processes, it is a challenging feedstock for further applications. Therefore, it is often burned in order to generate heat for the process itself in industrial processes [7]. To reduce the degradation of lignin by depolymerization and re-condensation during a pulping process, different attempts have been investigated that aim at using milder conditions to avoid lignin degradation. Examples for such processes are Organosolv-like processes, using organic solvents (e.g., ethanol, acetone) in combinations with diluted acid (e.g., sulfuric acid), liquid hot water pretreatments, which treats the biomass with water at elevated temperatures, or mild steam explosion, induced by sudden pressure release of heated wet biomass [3,8,9]. An alternative, which was developed in recent years, is the OrganoCat process. The OrganoCat process uses a biphasic solvent system as depicted in Scheme 1, consisting of an aqueous phase, which contains an organic acid to depolymerize the hemicelluloses, and 2-methyltetrahydrofuran (2-MTHF) as the organic phase, which is used to extract lignin *in-situ*. This way, the number of side reactions and alteration of the lignin is limited during the process [10,11,12]. However, lignin is still affected by the process and the number of oxygen-containing linkages is reduced, and new C–C linkages can be formed due to condensation reactions. As many strategies on lignin utilization focus on cleaving these oxygen containing linkages (mainly β-*O*-4), it is crucial to produce a feedstock that is rich in this type of linkages [13,14,15].

A strategy to provide potentially high value lignins is their fractionation from existing pulping processes. Lignin with different properties can be obtained, which should provide improved efficiency in the subsequent processes. Different strategies to fractionate lignin have been discussed recently, aiming to increase lignin homogeneity and improving its utilization in high value applications [16,17,18]. A feasible process for the separation of lignin from the OrganoCat 2-MTHF phase is antisolvent precipitation as described by Holtz et al. using *n*-hexane or *n*-pentane as antisolvent [19]. With this strategy, lignin can also be collected in fractions of different molecular properties from the OrganoCat product stream.

This work elucidates further the strategy to separate OrganoCat lignin dissolved in 2-MTHF on the basis of antisolvent precipitation and simultaneous fractionation. Solubility curves of lignin in a mixture of antisolvent and 2-MTHF were determined and different fractions of precipitated lignin were collected. The lignin fractions were then characterized via 2D-^1^H-^13^C-HSQC (heteronuclear single quantum coherence spectroscopy) NMR to determine the relative molecular size, the ratio of **S**-, **G**- and **H**-units, and the type and number of linkages between the different monomer units. This combined information is then used to give insight into the properties of the isolated OrganoCat lignin (OCL) fractions.

## 2. Results and Discussion

### 2.1. Solubility of Lignin and Furfural in n-Hexane/2-MTHF and n-Pentane/2-MTHF

**Figure 1:**
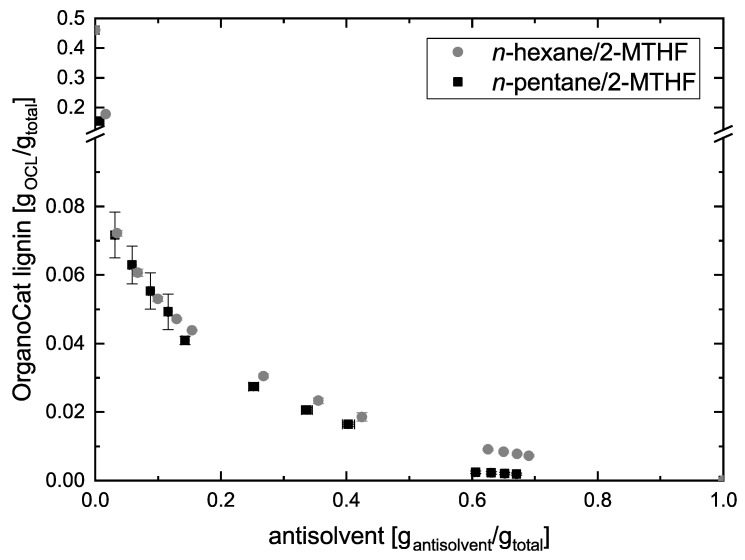
Mass fraction of lignin in equilibrium solution of 2-MTHF as a function of the mass fraction of antisolvent at ambient conditions. Black: OrganoCat lignin (OCL) solubility curve for *n*-pentane. Gray: OCL solubility curve for *n*-hexane; two sets of experiments for *n*-hexane; three sets of experiments for *n*-pentane.

The solubility of OrganoCat lignin (OCL) in the solvent system *n*-hexane/2-MTHF or *n*-pentane/2-MTHF, respectively, was determined using pure solvents, following the procedure described by Holtz et al. [19]. As reported, with increasing antisolvent-to-2-MTHF ratio the solubility of lignin decreases, as lignin is almost insoluble in pure *n*-hexane and *n*-pentane (see Figure 1). The *n*-pentane/2-MTHF mixture shows lower solubility of OCL than *n*-hexane/2-MTHF at similar antisolvent-to-2-MTHF ratios.

Furfural, obtained from degradation of pentoses in the OrganoCat process [10], is a main contaminant in the lignin solution. It was quantified in the antisolvent/2-MTHF solution, using gas chromatography with *n*-decane as the internal standard. The solubility of furfural is high enough in an antisolvent/2-MTHF mixture of a 1:1 volume ratio, for both antisolvents, to dissolve 1 wt% of furfural, which is comparable to concentrations obtained after OrganoCat pulping [10]. If a synthetic mixture of 10 wt% OrganoCat lignin and 1 wt% furfural is precipitated with *n*-hexane, over 80% of the furfural stays in solution. The rest precipitates together with the lignin. In the case of *n*-pentane, over 90% of the furfural is still in solution. The results show that this antisolvent system is not only capable of separating lignin from the 2-MTHF phase but also of separating lignin from furfural, further purifying the obtained lignin fractions. Isolation of furfural from the solvents and residual lignin fragments can be achieved straightforwardly by distillation. Furfural is an interesting platform chemical [20,21,22]; therefore, the separation of furfural from product streams is important to holistically valorize lignocellulosic biomass in a biorefinery and may add economic value, as it can be converted into other valuable compounds.

### 2.2. Size Distribution of Lignin Fractions

**Figure 2:**
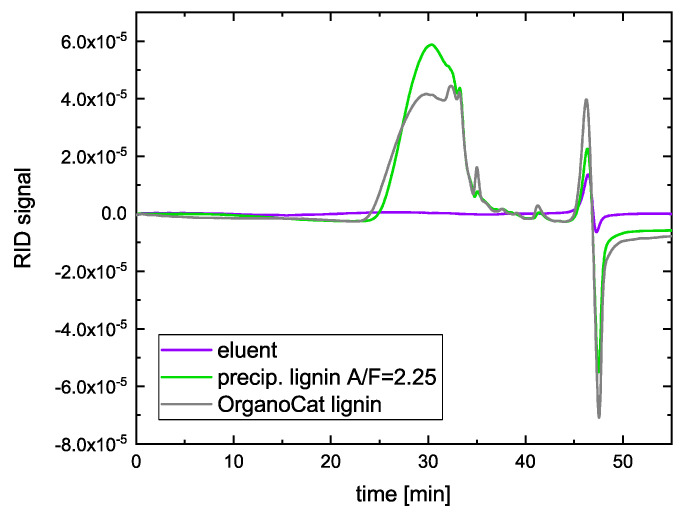
Size exclusion chromatography (SEC) chromatograms for OrganoCat lignin before and after precipitation from a 10 wt% solution in 2-MTHF with *n*-hexane. Eluent flow rate at 1 mL min^−1^. Lignin was precipitated from the crude solution using an *n*-hexane/feed volume ratio (A/F) of 2.25.

The molecular size distribution of different lignin samples was analyzed using size exclusion chromatography (SEC). In Figure 2, the SEC chromatograms for OrganoCat lignin (OCL) before and after precipitation with *n*-hexane are compared.

As smaller molecules eluate slower than larger molecules, the positive and negative peaks at 46 and 47 min, respectively, can be allocated to lithium chloride in the eluent. The chromatogram of OCL shows a broad signal starting at 24 min and ending at 38 min. An additional peak is observed at a retention time of 41 min. The broad signal in the OCL chromatogram can be explained by the heterogeneity of lignin. OCL is composed of molecules of various sizes, which results in a broad range of retention times for the different components. The additional peak at 41 min can be attributed to an OrganoCat process product that is significantly smaller than the other components, including molecules like furfural, aromatic monomers, or residual sugars, which are partially extracted into the organic phase during the OrganoCat process and can be observed in the NMR spectra. The chromatogram for lignin, precipitated with *n*-hexane at an antisolvent–feed ratio (A/F) of 2.25, shows a similar trend of the Refractive Index Detector (RID) signal. Based on the similarity of the chromatograms, the molecular size distribution and potentially the structure of lignin appear to remain virtually unchanged in the precipitation process.

The yield of precipitated lignin depends on the amount of antisolvent added to the feed solution (Figure 1). If only low amounts of antisolvent are added, lignin precipitates only partially. If more antisolvent is added, more lignin constituents are eliminated from the organic solution.

**Figure 3:**
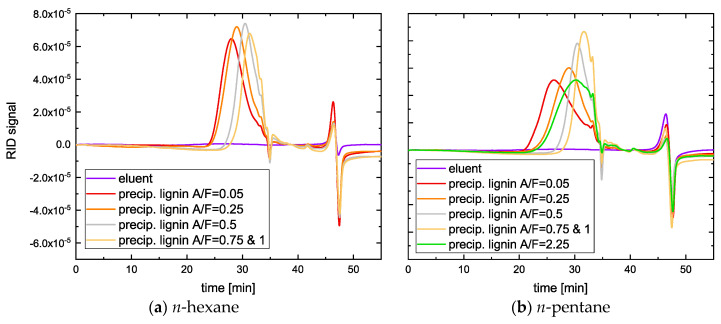
SEC chromatograms for 10 wt% OCL after consecutive precipitation using different antisolvent/feed volume ratios (A/F = 0.05–1) of (**a**) *n*-hexane and (**b**) *n*-pentane. In the case of *n*-pentane as the antisolvent, the A/F = 2.25 was precipitated in a separate experiment from a 10 wt% OCL solution.

It becomes apparent that with varying antisolvent/2-MTHF ratios different lignin fractions are precipitated (Figure 3). When lower amounts of antisolvent are added, larger lignin constituents are precipitated from the organic phase, whereas with higher amounts, smaller lignin constituents are precipitated. These results are in accordance with a previous study showing that the molar mass distribution of precipitated Kraft lignin fractions depends on the amount of antisolvent (water) added in the precipitation step [23].

Similar trends for sample precipitation are observed for *n*-pentane as for *n*-hexane. However, for *n*-pentane the difference in size between the obtained fractions is larger compared to *n*-hexane. Maxima of the size distribution curves for *n*-pentane range from 26.2 min at A/F ratio 0.05 to 31.7 min at A/F ratio 0.75 and 1. In the case of *n*-hexane, the maxima of these fractions are at 28.0 min and 31.3 min, respectively. The average *Δ*t between two fractions with *n*-pentane as antisolvent is 1.8 min compared to 1.1 min for *n*-hexane, indicating that there is a considerable size difference of the obtained fractions due the different solubility of lignin in *n*-pentane than in *n*-hexane.

By the fractional precipitation the heterogeneity of the lignin can be reduced. This displays a promising approach to deliver lignin suited for more specific applications. Compared to fractionation methods already reported in the literature [23,24,25], the use of a single antisolvent is beneficial, as the effort for the recovery of the antisolvent is reduced.

### 2.3. Chemical Composition of Lignin Fractions

To evaluate possible differences in the molecular composition of the different lignin fractions, 2D-^1^H-^13^C-HSQC-NMR spectroscopy was used to determine **S**-, **G**-, and **H**-units as well as main linkages between the units. The signals observed in the NMR spectrum were referenced using literature known values for the respective substructures [26,27]. The signal intensities for **S**-, **G**-, and **H**-units were determined identically via 2D-^1^H-^13^C-HSQC-NMR spectroscopy for each spectrum and added up to give the total number of units in the respective fraction. This way, the unit composition of each fraction could be calculated. Additionally, the three main linkages between the units, β-*O*-4, β-β, and β-5 were quantified accordingly.

**Figure 4:**
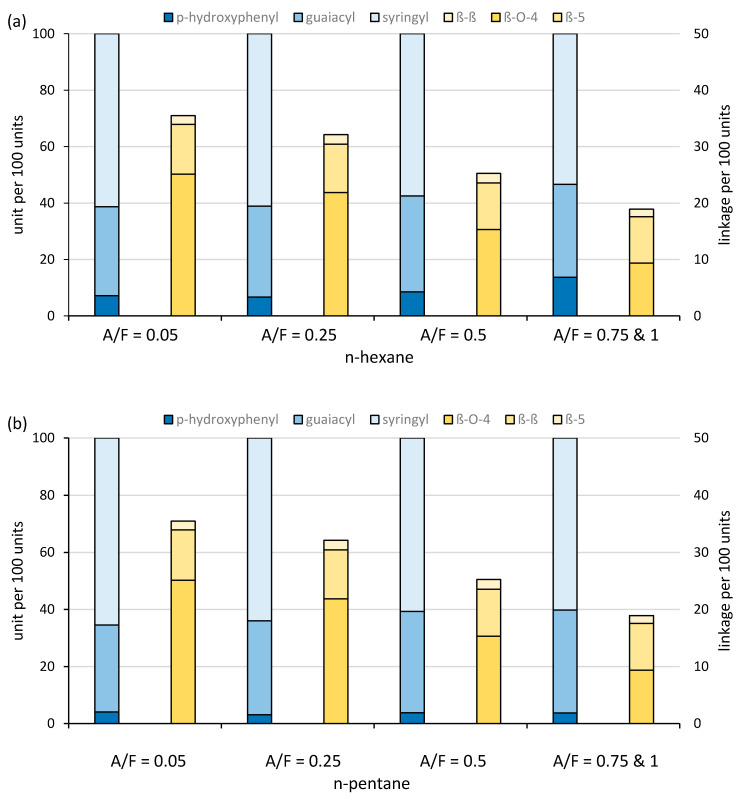
Lignin unit and linkage distribution obtained from ^1^H-^13^C-HSQC NMR results for OCL after consecutive precipitation using different antisolvent/feed ratios (A/F) of (**a**) *n*-hexane and (**b**) *n*-pentane. (For expansion of HSQC-NMR spectrum see Figure S1 in Supplementary Materials)

Figure 4 shows the NMR results for the fractionation of lignin using *n*-hexane (a) and *n*-pentane (b) as antisolvents at the same antisolvent to feed ratios as investigated previously via SEC. The composition of **S**-, **G**-, and **H**-units does not change significantly throughout the fractions. This leads to the conclusion that the monomer units in the polymeric structure of the used woody material are distributed statistically, which is in accordance with the findings of Metzger et al. [28], who showed that, in wood lignin, even at smaller oligomer stages the monomer units are statistically distributed.

The number of β-β and β-5 linkages per monomer unit in the different lignin size fractions does not change. In both types of linkage, the two monomer units are connected via a comparatively stable C–C bond. Due to the mild reaction conditions, the C–C bonds—being more stable than C–O bonds—are not expected to be cleaved during the pretreatment. In contrast, the β-*O*-4 linkages are partially cleaved under the given pretreatment conditions [29]. With increasing molecular weight of the different fractions, the number of β-*O*-4 linkages decreases, showing that the main reason for depolymerization of lignin during the pretreatment is caused by cleaving β-*O*-4 linkages between monomer units (Figure 4). In contrast to the trends observed in SEC measurements, no significant difference in the composition of lignins precipitated with *n*-hexane or *n*-pentane could be observed. This might indicate that the fractions do vary in size; however, the chemical composition of the polymer chains is similar.

It is apparent that the main driver for lignin precipitation is the change in polarity of the solvent system after addition of the antisolvent. *n*-hexane and *n*-pentane have similar polarities, both drastically lower than the polarity of 2-MTHF, thus decreasing the overall polarity of the antisolvent/2-MTHF mixture upon addition of further antisolvent [30,31]. Holtz et al. reported that the optimal antisolvent regarding overall process efficiency is *n*-pentane [19]. Results obtained for *n*-pentane show a wider difference in size of the obtained fractions and similar chemical compositions compared to fractions acquired using *n*-hexane. This makes it indeed a very promising candidate for lignin fractionation. It enables the preparation of lignin fractions with different sizes and linkage networks, potentially improving the follow-up utilization of lignin due to an increased homogeneity.

## 3. Materials and Methods

### 3.1. Materials

*n*-Pentane (>99%), *n*-hexane (>99%), and 2-methyltetrahydrofuran (2-MTHF, >99%) were obtained from Carl Roth, and furfural (>98%) was obtained from Alfa Aesar. All chemicals were used without further purification. Beech wood (*Fagus sp.*) was obtained from local suppliers; the particle size was generated by a cutting mill with a 10 mm sieve and dried at 50 °C until weight constancy (ca. 24 h), leaving a residual moisture content of approx. 10% water.

### 3.2. Procedure for OrganoCat Pulping

In a 300 mL Parr high pressure reactor, 12 g beech wood (*Fagus sp.*) lignocellulose, 120 mL ultrapure water, 1.08 g oxalic acid, and 120 mL 2-MTHF were mixed and stirred at 140 °C for 3 h. After cooling the reactor down to room temperature, the liquid phases were separated by decantation. The residual oxalic acid in the organic phase was precipitated adding 60 mL of a 0.2 M solution of CaCl_2_ in water and stirring for 20 h. After phase separation and filtration of the solid residue, OrganoCat lignin (OCL, M_W_ = 2573 g mol^−1^) [10] was obtained by evaporation of 2-MTHF at reduced pressure.

### 3.3. Procedure for Lignin Fractionation

Following the procedure by Holtz et al. [19], a solution of 10 wt% lignin in 2-MTHF was prepared by addition of OCL to 2-MTHF. The solution was mixed on a laboratory shaker (MKR23 Hettich Lab Technology, Tuttlingen, Germany, *n* = 400 rpm) at 40 °C for 10 min. Insoluble lignin particles were separated using a centrifuge at *n* = 3000 rpm at 25 °C for 10 min. The solution was filtrated. Lignin was precipitated by addition of the respective antisolvent in several consecutive steps, except for A/F = 2.25, which was directly precipitated in a separate experiment. After each addition of antisolvent to the lignin/2-MTHF solution, the precipitate was centrifuged and filtered. A maximum of five consecutive precipitation steps was performed. Precipitated lignin was dried in a vacuum oven for 24 h at 40 °C and 10 mbar absolute pressure and weighed.

### 3.4. Lignin Analysis (NMR spectroscopy)

NMR measurements were conducted on a Bruker AS400 (400 MHz) Spectrometer (Billerica, MA, USA). Approximately 50 mg of lignin was dissolved in 0.5 mL of DMSO-*d*_6_. ^1^H-^13^C-HSQC (measurement time 3:40 h) measurements were used to evaluate the type of linkages present in the respective lignin fractions of the reactions. The corresponding signals of each substructure were integrated and quantified using mesitylene as the internal standard.

### 3.5. Lignin Analysis (SEC)

The molecular size distribution of lignin samples was investigated using size exclusion chromatography (SEC). Measurements were performed using an Agilent Series 1200 SEC device equipped with an RID detector (Optilab Rex 837, Wyatt Technology, Santa Barbara, CA, USA) and a SEC column system consisting of a precolumn PSS PolarSil, 8 × 50 mm, with a particle size of 5 μm, and three columns of PSS PolarSil Linear S, 8 × 300 mm, particle size μm downstream. SEC measurements were conducted using *N*,*N*-dimethylformamide with 0.1 M lithium chloride as eluent, a column temperature of 45 °C, and a flow rate of 1 mL min^−1^. Samples of lignin with concentrations of 30 g L^−1^ were dissolved in the eluent. The injection volume used was 10 μL.

### 3.6. Furfural Quantification (GC)

GC measurements to quantify the amount of furfural in the 2-MTHF phase were conducted on a Thermo Scientific Focus Gas Chromatograph using a 30 m HP-Innowax column, with helium as the carrier gas with a flow rate of 1.5 mL min^−1^ and a flame-ionization detector (Thermo Scientific, Waltham, MA, USA). The initial temperature was 50 °C, raised by 8 °C min^−1^ to 250 °C, and kept at 250 °C for 5 min. Quantification was done using *n*-decane as the internal standard.

## 4. Conclusions

Lignin obtained from OrganoCat pretreatment has been precipitated and fractionated successfully. The resulting fractions have been analyzed using SEC and 2D NMR techniques in order to elucidate the structure of the different fractions. OrganoCat lignin (OCL) has been fractionated into larger and smaller molecular weight distribution fractions. The larger molecules contained more β-*O*-4 linkages per monomer unit than the smaller fractions. β-β and β-5 linkages did not change, as the required process conditions are not met to cleave these types of bonds. The monomer unit composition does not vary between the fractions, indicating that the lignin polymer is statistically distributed. This fractionation procedure might trigger further applications of lignin fractions and potentially increase the efficiency of existing lignin conversion processes, due to the enhanced homogeneity of the obtained lignin fractions.

## Supplementary Materials

The following are available online, Figure S1: Expansion of HSQC NMR spectrum of OrganoCat lignin.
