# Supplementary material for: Lignin Precipitation and Fractionation from OrganoCat Pulping to Obtain Lignin with Different Sizes and Chemical Composition
Source: Molecules. 2020 Jul 22;25(15):3330. doi: 10.3390/molecules25153330 (PMC7436272; doi:10.3390/molecules25153330)
Supplement: Supplementary file 1 [file molecules-25-03330-s001.pdf]

Supporting Materials

Lignin Precipitation and Fractionation from OrganoCat Pulping to Obtain Lignin with Different Sizes and Chemical Composition

Dennis Weidener [1,2,3], Arne Holtz [3,4], Holger Klose [1,3,5], Andreas Jupke [4], Walter Leitner [2,6] and Philipp M. Grande [1,3,*]

1 Institute of Bio- and Geosciences, Plant Sciences, Forschungszentrum Jülich GmbH, 52425 Jülich, Germany
2 Institute of Technical and Macromolecular Chemistry (ITMC), RWTH Aachen University, Worringer Weg 1, 52074 Aachen, Germany
3 Bioeconomy Science Center (BioSC), c/o Forschungszentrum Jülich, 52425 Jülich, Germany
4 Fluid Process Engineering (AVT.FVT), RWTH Aachen University, Forckenbeckstraße 51, 52074 Aachen, Germany
5 Institute of Biology I, RWTH Aachen University, Worringer Weg 3, 52074 Aachen, Germany
6 Max-Planck-Institute of Chemical Energy Conversion, Stiftstraße 34-36, 45470 Mülheim an der Ruhr, Germany
* Correspondence: p.grande@fz-juelich.de; Tel.: +49-2461-616341; Fax: +49-2461-612492

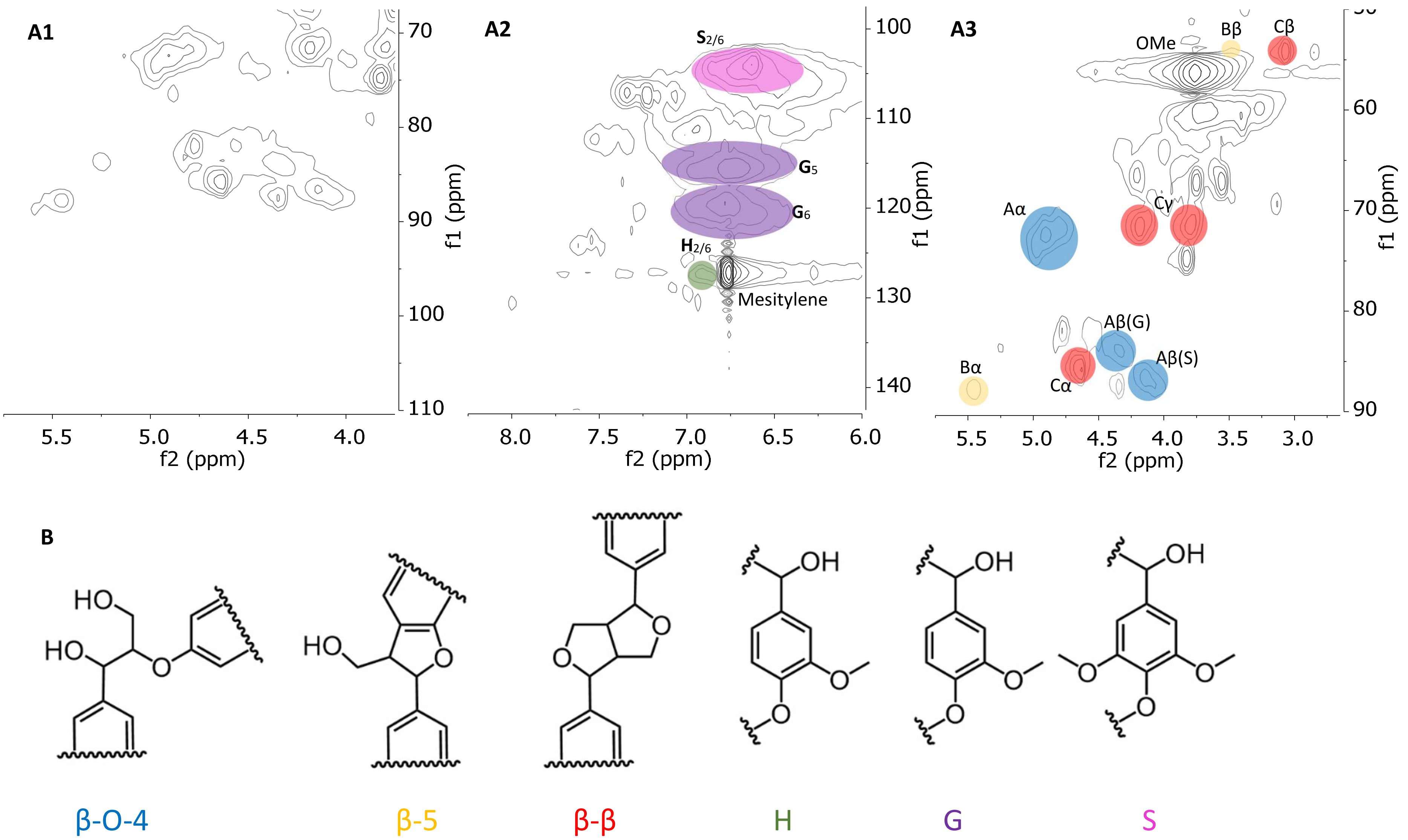

Figure S1: Expansion of HSQC NMR spectrum of OrganoCat lignin, A1 (carbohydrate region), A2 (lignin region) and A3 (aliphatic region), B investigated linkages and monomer units.
